# Liberalizing abortion to reduce maternal mortality: expanding access to all Ethiopians

**DOI:** 10.1186/s12978-022-01457-z

**Published:** 2022-06-27

**Authors:** Mekdes Daba Feyssa, Saba Kidanemariam Gebru

**Affiliations:** 1 St. Paul’s Hospital Millennium Medical College, Addis Ababa, Ethiopia; 2 Center for International Reproductive Health Training, Addis Ababa, Ethiopia; 3 Global Consultant for Sexual and Reproductive Health and Rights, Rockville, MD USA

**Keywords:** Safe abortion, Abortion policy, Ethiopia, Abortion law

With a goal of reducing maternal mortality and morbidity, Ethiopia liberalized its abortion law in 2005, making it one of the early champions in Africa of expanding access to safe abortion services [1]. Prior to revising the abortion law text within the Revised Criminal Code of the Federal Democratic Republic of Ethiopia, which had previously only allowed abortion to save a woman’s life, the country had one of the highest maternal mortality rates in the world. Estimated at 687 maternal deaths per 100,000 live births in 2005, one-third of these maternal deaths were attributed to complications related to unsafe abortion [1, 2].

The revised abortion law permits abortion to preserve a woman's health in the case of rape, incest, and fetal impairment [3, 4]. In addition, pregnancy termination is permitted for those with physical or mental disabilities and/or minors who may be physically or mentally unprepared for childbirth [3]. Importantly, the law states that a woman’s word is sufficient evidence of rape or incest and that stated age is all that is needed to allow an age-based abortion. Within a decade of liberalizing the penal code, the proportion of maternal deaths attributable to unsafe abortion dropped from 32% to less than 10% [5].

The law’s revision was spearheaded by a coalition of Ethiopians representing the medical, political, and women’s rights communities. These groups joined forces to produce timely data and present evidence to decision makers on the health and social consequences of unsafe abortion by outlining ways to reduce maternal mortality and fulfill the rights outlined in the country’s constitution, particularly Article 35 [6, 7]. Strong government support for global human rights conventions, including the Convention on the Elimination of All Forms of Discrimination Against Women [8] and the Protocol to the African Charter on Human and Peoples’ Rights on the Rights of Women in Africa, also known as the Maputo Protocol [9], provided a further foundation to the penal code revisions.

Soon after the penal code was liberalized, the Ministry of Health launched a bold implementation program to guide the introduction and scaling up of safe abortion services. These included publishing comprehensive national standards and guidelines in 2006 that outlined clinical norms and set the stage for an expanded scope of practice for multiple cadres of health care workers, including health extension workers, provision of competency-based training, and supportive supervision for providers [6]. These efforts have been successful due in part to the strong national-level advocacy and technical leadership of Ethiopian civil society organizations, such as the Ethiopian Society of Obstetricians and Gynecologists, the Ethiopian Women Lawyers Association, and the Consortium of Reproductive Health Associations.

However, achieving abortion access for all remains a challenge. Although the proportion of abortions performed in health facilities increased from one-quarter to more than half between 2008 and 2014, the disparity in access between urban and rural settings persists [10]. Further, despite nearly two decades since the passage of the law, public knowledge about the legal status of abortion remains limited, as is evidenced by data from several knowledge and attitudinal surveys. One survey conducted in Arba Minch, in the Southern region, found that only one-quarter of respondents were aware that abortion is legal and another, in Bahir Dar, in the North-West of the country, reported that two-thirds of respondents knew that abortion was legal in some circumstances [11, 12]. The stigma around abortion persists and impacts both providers as well as those seeking safe abortion care. A 2015 study of provider attitudes towards safe abortion in Addis Ababa found that while three-quarters of those surveyed were aware of the provisions of the abortion law, they did not necessarily support them [13]. Seven out of 10 respondents said that they were not comfortable working in a place where abortion is performed given their personal religious values, lacked training on the method, and/or considered abortion to be outside of the scope of their practice [13]. Abortion providers often experience disapproval and isolation from their peers, which discourages them from continuing to provide the service. Poor understanding of the law, mistreatment, and denial of services associated with providers’ personal beliefs can still result in individuals seeking unsafe abortion outside of the formal health care system. Work still needs to be done to understand how abortion stigma manifests at health facilities and within various communities to improve existing stigma reduction interventions, such as values clarification and attitudinal transformation and Providers Share Workshops [14–17].

Progress toward abortion access in Ethiopia is stifled by the changing global landscape of sexual and reproductive health (SRH) and rights, including the impact of the United States’ recurring Global Gag Rule (GGR), which has required foreign nongovernmental organizations (NGOs) receiving U.S. government funding to agree not to perform or promote abortion. NGOs, including Marie Stopes International and the Family Guidance Association of Ethiopia, were obligated to close some clinics in Ethiopia due to their refusal to compromise their clients’ rights to full access to SRH services [18]. It will take some time for SRH services to scale back up to their previous footprint throughout the country, now that the GGR has again been reversed. However it is likely the GGR will be implemented again in the future, threatening the provision of safe abortion services. The recovery of SRH services has been doubly hampered by the onset of COVID-19 as well as both humanitarian crises and natural disasters, resulting in an increase of internally displaced persons. Our shared lived experience of the COVID-19 pandemic illustrates how quickly mobility and access to services can be limited and provides yet one more example to why increasing access to abortion through a range of modalities is crucial to reproductive autonomy. Now more than ever, it is time to complete the country’s shift from unsafe to safe abortion and to incorporate new models of service delivery that are person centered. De-medicalizing medical abortion offers several potential avenues to increase access including self-administration of medical abortion pills through telemedicine [19], community-trained health workers [20, 21], and pharmacies [22, 23]. In January 2021, the government of Ethiopia started reviewing a new guideline for the self-use of abortion medications in some circumstances [24]. This draft guideline will set the stage for organizations, advocates, and other community actors to further increase access to abortion.

Simply stated, more efforts are needed to ensure that, when they need it, all women and girls in Ethiopia can access quality abortion in a welcoming and supportive environment. Efforts to integrate the provision of abortion services into general maternal care, should be strengthened. While safe abortion services should be provided regardless of a woman’s ability to pay, declining global funding and assistance requires that Ethiopia explore diversified domestic financing options, for example through including abortion in the community-based health insurance. Addressing stigma in providers as well as those seeking care and increasing the pool of abortion providers by exploring new avenues for delivery of abortion care are paramount. Health care professionals and advocates must continue to work together to hold the government accountable for normalizing abortion care and support its full integration into the country’s maternal health program by including it in preservice training, making the service free of charge, and including it in essential health care services. In so doing, stakeholders should continue to embrace a rights-based approach that aligns with the spirit of achieving universal access to health care as ascribed in Sustainable Development Goal 3.7 [25].

## Data Availability

Not applicable.

## Abbreviations

GGR: Global Gag Rule
NGO: Nongovernmental organizations
SRH: Sexual and reproductive health
